# Anticipatory governance for newcomers: lessons learned from the UK, the Netherlands, Finland, and Korea

**DOI:** 10.1186/s40309-021-00179-y

**Published:** 2021-07-11

**Authors:** Kyungmoo Heo, Yongseok Seo

**Affiliations:** Moon Soul Graduate School of Future Strategy, Korea Advanced Institute of Science and Technology (KAIST), N5 #2237, Daehakro 291, Yousung, Daejeon, Republic of Korea 34170

**Keywords:** Anticipatory governance, Capacity building, Future receptivity, Newcomer, Public participation

## Abstract

Anticipatory governance (AG) is defined as a “system of systems” that employs foresight to create future plans and execute relevant actions. Recently, various frameworks of AG have been introduced, but there is little practical information available for newcomers on how to do this. This research conducted a framework-based comparative country analysis to provide lessons learned for newcomers in the sphere of foresight-linked AG. By evaluating the AG levels of Finland, the UK, the Netherlands, and Korea, we found that the consequences of foresight-linked AGs were different in each country. At the same time, we also identified a common denominator, namely, *future receptivity*, a “human or people” capacity to accept and understand the value of foresight. Instead of temporary system changes or organizational modifications*, future receptivity* is an underlying element for newcomers to overcome lingering short-termism and facilitate the coordination of stakeholders concerning foresight. In conclusion, we suggest ways to promote *future receptivity* for newcomers. First, the government should educate and train the public and government officials to promote *future literacy and future proficiency*. Second, the government should provide a process for public participation such as nationwide networking that enables the public to influence their diverse future images over foresight outcomes.

## Introduction

concept of anticipatory governance (AG) has grown in academic and practical repute in the “futures” sphere since Fuerth and Faber suggested it be applied to the US administration in 2012 [1]. For the then US administration, AG meant anticipating emerging issues and trends and evaluating the implications and impact of government policies under different circumstances. Besides the USA, various countries have introduced AG into their government affairs [2–6]. Moreover, in international organizations, AG stands out too. UNDP’s Foresight Manual was published in 2018 [7], which outlines “Anticipatory Governance and Strategic Management” as one of the four major areas where foresight can make an important contribution to the work of public bureaucracies as part of the implementation of the UN’s Sustainable Development Goals. Subsequently in 2019, the Organization for Economic Cooperation and Development (OECD) hosted the 38th Session of the Network of Senior Officials from the Centres of Government to discuss AG application within the Prime Minister’s Office [8].

Various frameworks have been introduced as practical tools for developing a foresight-linked AG diagnosing where and how certain AG approaches may need improvement or refinement [9–11]. The literature review reveals that there is much information available about foresight methodologies, processes, and rationales for AG. However, there is little concerning underlying factors that can lead to the success of AG. Rather, more focus is on operational factors which appear to merely determine whether foresight exists or not. Furthermore, there is little practical information on AG for new foresight practitioners and countries pursuing the use thereof, namely, newcomers. The AG framework along with its delivery and reporting methodologies has been similar around the world. It has been developed mainly by AG leading countries [11]. Most papers do not attempt to look at AG through the eyes of newcomers. Indeed, it is necessary to delve deeper into historical and ongoing efforts (or best practices) of countries that lead foresight-linked AG. In addition, an analysis of where and why countries have lost the functioning a foresight-linked AG is also critical for newcomers for benchmarking purposes.

This article attempts to address the following question: for the continued foresight and sustainable operation of AG, what kinds of fundamental conditions (capabilities and systems) are needed for newcomers? The objective of this article is to explore the concept of knowledge that initiates and continues AG from the perspective of newcomers. It is natural for some to regard that the implementation of AG should be different for newcomers especially those that do not have a long-established history of democracy, solid social stability, and a certain degree of welfare. For newcomers who plan to initiate AG, it is important to identify a fundamental starting point and sustaining conditions while exploring specific implications in the sphere of AG.

The structure of this article is as follows. First, it presents the theoretical frames and methodology used in the study. Second, it proposes a new framework designed by integrating the AG criteria found in the key AG literature with Fuerth and Faber’s frame. On the basis of this framework, the AG capabilities and systems of the UK, the Netherlands, Finland, and South Korea (hereinafter Korea) are evaluated and compared. Lastly, based on these findings, this article concludes with recommendations for a continued foresight-linked AG for newcomers.

## Theoretical frames and methodology

### Futures studies, horizon scanning, foresight, and anticipatory governance

Futures studies, horizon scanning, foresight, and AG all explore the realm of “futures,” but their range and purposes are slightly different. Futures studies is the academic domain and theoretical backbone of horizon scanning, foresight, and AG. It focuses on values and norms including alternative ideas, systems, and models rather than policies [12–14]. Many studies in this sphere entail images, worldviews, behaviors, and advances in the science and technology of various futures of both society and people in general [12]. Meanwhile, foresight rather intends to influence policies and strategies through forecasts and estimations while emphasizing forward-looking outcomes within government policies [15–17]. In short, while futures studies aim to cope with and embrace uncertainty, foresight focuses more on eliminating it.

**Fig. 1:**
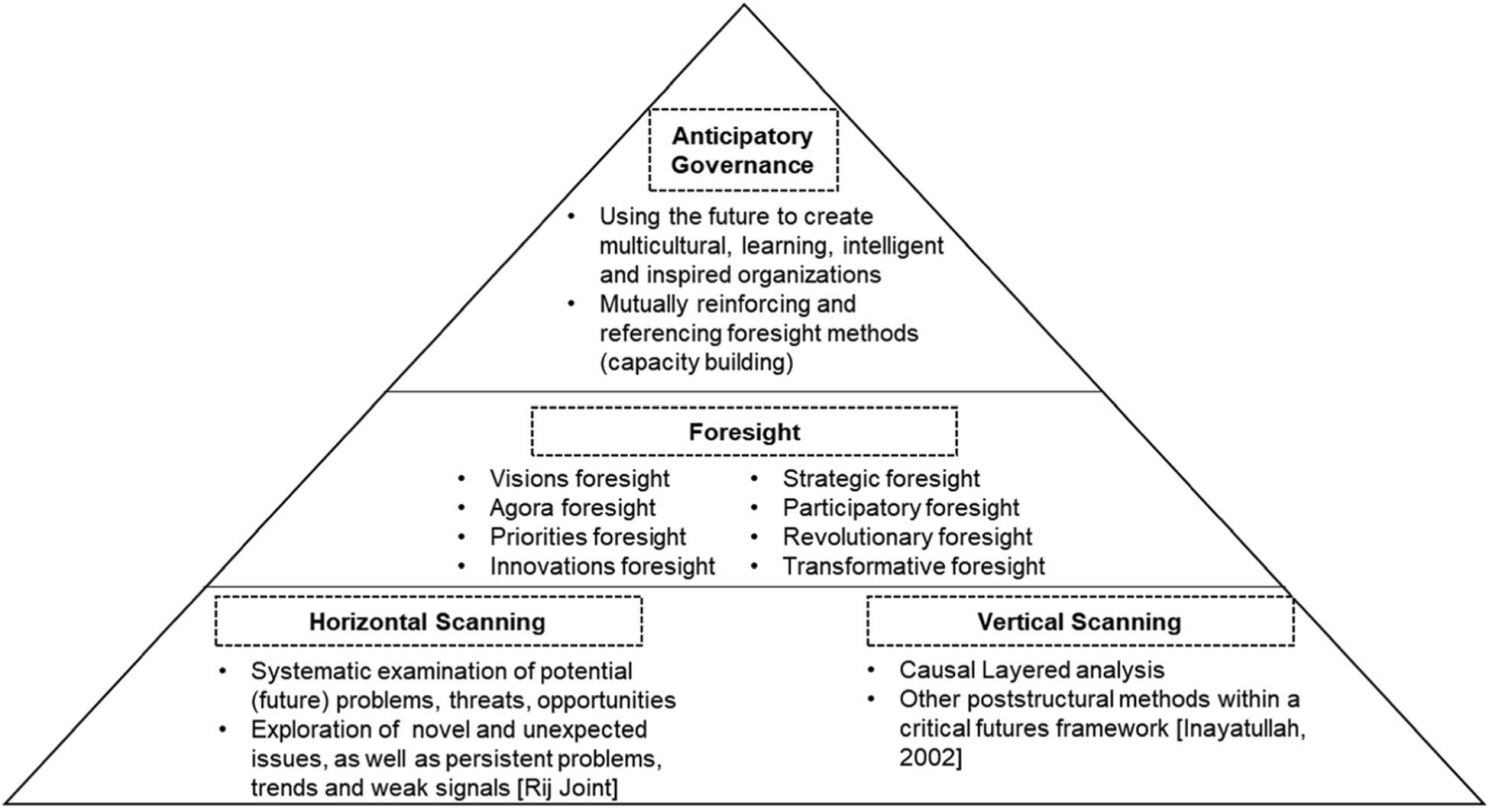
Vertical layers of horizon scanning, foresight, and the AG

Horizon scanning is the foundation and an essential feedstock of integrated foresight [18]. It is a fundamental approach to most of the “more specific focused” foresight exercises [19]. At the beginning of any foresight activities, horizon scanning is implemented as a prerequisite [20]. In short, horizon scanning is a broad concept consisting of forward-looking activities [21]. It can sometimes be used as a foresight tool, a special form of foresight, or a broad scope foresight [22]. For example, Korea and Finland use horizon scanning as a specific and indispensable tool for foresight and AG [23], but the UK and the Netherlands use it similar to an entire foresight process. In the UK, horizon scanning is applied as a future developmental plan while improving policy robustness and detecting gaps in the knowledge agenda of government affairs [19]. In fact, horizon scanning is utilized as to roughly identify future issues “that need our attention in a systemic way to direct the future in a more desirable direction after a participative process of thinking and debating” [22]. Figure 1 explains the vertical layers of the above concepts—futures studies, horizon scanning, foresight, and AG.

This article narrows down the focus of the research to AG. It is true that horizon scanning and foresight are fundamental to AG. Indeed, they are broader domains of AG [14]. AG becomes one type of various approaches of foresight, namely, strategic (priorities) foresight or participatory (agora) foresight [24, 25]. For the purpose of this article, however, the “capacity” and “system” aspects of government foresight is emphasized, namely, a foresight-linked AG.

### Meaning and development of anticipatory governance

AG comes from a combination of (1) governance—decision-making rules and processes which determine stakeholders’ rights and responsibilities through coordination [26–28] and (2) anticipatory—organization or individual capacity “to deal with new situations and realize accepted values, changing the focus from forecasting to being ready for future challenges” [29, 30]. In short, AG can be defined as a sustainable decision-making process based on consensus on a desirable future or vision through the participation of various stakeholders including government, market, the public, and academics.

In terms of government affairs, Fuerth and Faber define AG as a “system of systems” [1]. AG employs foresight to create future plans and execute actions and utilizes foresight as a tool or process for enabling governments to deal with accelerating and complex forms of changes [9, 31]. Put differently, foresight-linked AG seeks to integrate foresight to respond to blunt threats at the early stages of development while focusing on increasing the capacity to act upon a wide range of possible futures with flexibility and adaptability. Therefore, it requires broader knowledge about future-oriented governance, which enables forward-looking decisions within institutions, ensuring stakeholders make more informed and wiser policymaking decisions [32].

Various man-made political and social institutions have tried to overcome the notion of complexity and have made plans looking beyond distant horizons; however, unexpected and hard-to-predict events often limit our attempts to glimpse into the future. To push through the above impasse, AG was introduced to government affairs in the 1990s by Osborne [33]. It was born as a repercussion of traditional predict-and-plan approaches and newly introduced planning tools including long-term planning and scenario and strategic planning [32, 34]. However, at that time, a concept of AG was also another form of new public management, connected with neoliberalism (running a government like a private enterprise) rather than depicting future-oriented strategies in governance [32]. It took some time for AG to build and convey the meaning acknowledged above.

Globally, AG has been widely and practically applied to other academic domains such as e-government and knowledge management [35, 36]; international development [37, 38]; crisis prevention and violence mitigation in international conflict [39]; environment studies and policy, socio-ecology, climate change, and sustainable development [34]; and anthropology with regard to using different perspectives, theories, models, and methods of anthropology in an anticipatory manner [40, 41]. In particular, AG has become a major topic of science and technology (S&T) studies such as emerging technologies, real-time technology assessment, and energy [32, 42, 43]. These applications to various academic fields are good examples of how AG is a suitable topic, concept, and practical method not only for policy and administrative studies but for other domains as well.

### Comparative case analysis based on a newly proposed framework

This article uses both an exploratory case study [44] and a framework-based comparative country analysis [45, 46]. First, the new framework is created based on Fuerth and Faber’s AG criteria complemented with other AG frameworks found in the literature. This framework is used as standardized criteria for a more comprehensive comparative case analysis between countries. Second, countries are compared based on the framework newly created for a comparative analysis. The choice of framework results in unique policy outcomes; therefore, comparing frameworks of countries can imply factors behind a country’s own distinctive success or failure [47, 48]. Particularly, this article selects and compares four different countries, each of which has distinctive historical and institutional features in AG development. From the results, it can be seen that foresight (namely, horizon scanning) has been losing its importance in the UK and the Netherlands since 2012 while Finland has successfully implemented foresight and achieved AG as an exemplary model for other countries to follow since the 1990s. In particular, Korea experienced a 40-year-long foresight deadlock and has only recently re-initiated a national foresight-linked AG. Third, the process and rationale for the exploratory case study is rather straightforward in this article structure. Taking into account the research questions, “why” and “how,” the exploratory case study would be more practical in providing detailed but underlying information and meaningful implications that this article delves into [49, 50]. This approach tends to focus on some representative examples of institutional measures, followed by a summarization of historical differences between countries [51, 52]. Fourth, to achieve validity and reliability, data are collected mostly by qualitative methods such as literature analysis, meeting materials, and a review of various government reports (including internal ones). Beside these methods, various sources of information including academic and professional experiences of the authors, experts’ opinions, and internal and official communications are included to enrich the case study further.

A few caveats regarding the methodology applied should be mentioned. It is inevitable that a framework-based comparison may be somewhat intuitive and rely on the viewpoints of experts. First, the assessment of AG can be rather partial and incomplete. However, all the topics covered here are too large and complex to fit into this short article. Assessing the quality of AG is part of the wider task of judging the overall quality of governments and public institutions. For example, both topics and elements of “anticipatory” can be attributed to good governance [11]. Second, assessments of the quality of AG involve an exercise of judgment that can be potentially biased. The above countries have different institutional setups including research infrastructures and styles of governance. Therefore, this article is not attempting to question the degree to which the prevailing political-foundational context is supportive. Rather, we wish to focus on the capacity and conditions of AG once the basic context has already (or mostly) been assured and is in place.

## Results

### A new framework as an analytical foundation

This section outlines a new framework based on Fuerth and Faber’s AG criteria [1]. To begin with, a number of frameworks along with their criteria taken from the key AG literature have been reviewed as seen below in Table 1. The literature selected is limited to the most relevant spheres in order to focus on foresight-linked AG in government affairs.

**Table 1:** Anticipatory governance framework with criteria in key literature

View on literature	AG criteria
Governance transform [53]	(a) Emerging trends oriented from current issues driven, (b) Long-term and strategic planning from short-term decision-making (c) Statewide plans with shared vision and goals from individual agency plans and objectives (d) Holistic approaches to crosscutting issues from piecemeal solution to immediate problems
System for the AG [9]	(a) Foresight system (b) Networked system for integrating foresight and the policy process, (c) Feedback system to gauge performance and also to manage “institutional” knowledge, (d) Open-minded institutional culture
Strategic design [54]	(a) Vision of preferred futures that resonate strongly with people’s values and aspirations, (b) Intelligence to identify critical emerging issues, (c) Strategy development to translate aspirations into realities
Assessing the quality of the AG [10]	(a) Planning processes and foresight, (b) Policy and regulatory framework, (c) Policy and regulatory framework, (d) Representation of future interests, (e) Performance measure and reporting (f) Resilience, risk reduction, and emergency management, (g) Mechanisms for problem solving and consensus building for long-term policy challenge
Science Technology studies [32, 42]	(a) Foresight as an anticipation and assessment of new and emerging technologies, (b) Engagement with the lay public to identify society concerns to R&D processing, (c) Empowerment and integration for creation of opportunities and further long-term reflective capacity building

For the new framework, this article combines Fuerth and Faber’s three criteria with other criteria taken from the above AG literature [1]. Fuerth was the structural founder of AG in government affairs. He applied AG to the Clinton administration in a practical, immediate, and time- and cost-efficient manner. He made a clear distinction with foresight explaining that AG is “a scalable system of systems” and “a system of institutions, rules and norm” [1]. Fuerth and Faber list three core criteria as the systemic approach to be taken when a government utilizes foresight in policymaking and implementation, namely, (1) foresight system, (2) networked system, and (3) feedback system [1].

Based on Fuerth’s notion of an “open-minded institutional culture” [9], this article introduces the idea of a “continuity system” as a new criterion. This concerns the meaning of organizational and individual capacity-building for continued foresight and the sustained operation of AG. Continuity system is derived from an in-depth review of the various AG criteria listed in Table 2. The original Fuerth and Faber framework attempts to overcome the inherent vagueness or limitations within the foresight process. They then introduced an organizational modification and a readjustment of government systems and decision-making structures. However, this practicability and applicability also reveals some limitations including the myopic view of the objective of AG and a lack of continuity due to a “one-time” implementation paternal practice. AG needs to be implemented on a long-term basis for review, evaluation, and refinement because foresight is about studying future-oriented perspectives and looking beyond the present. Rather than focusing on temporary systems or organizational modifications, a foresight-linked AG should maintain a sustainable operation by internalizing forwarding-looking attitudes and future-oriented minds. Without exaggeration, the continuity system assumes to be a precondition for initiating and continuing AG.

**Table 2:** New anticipatory governance framework with criteria

Criteria	Capability [C] and System [S]
Foresight system	[C] Gathering of long-range information; disciplined analysis, predicting future contingencies of interest, backcasting for vision, strategies, and actions, forward engagement [S] Influential and independent foresight entity to offer a protected space
Networked system	[C] Leadership with commitment, effective communication, inter-ministerial and regional coordination, integration of foresight and the policy process [S] Hub and spoke-based governance with networking integrator, venue for public participation
Feedback system	[C] Watch and audit, diagnosis with performance evaluation, trial-and-error, indicator checking, recalibration, organizational learning [S] Feedback mechanism and institutional knowledge management
Continuity system	[C] Internalization, open-minded institutional culture, anticipatory action learning, foresight receptivity with orientation to action, future fluency of professionals, future literacy for the public [S] Formal training and re-education process, repetitive and constant practice, academic and research institution

Table 2 depicts the new framework along with new criteria including the capabilities and systems for the further comparative analysis of countries. In the table, a system denotes a governance tool to achieve or even reinforce named capabilities. In the next section, based on this new integrated framework, the AG capabilities and systems of Finland, the UK, the Netherlands, and Korea are compared.

### Analysis of countries based on the new framework

#### Finland’s AG: role model

Against the backdrop of the economic crisis in 1990, the Finnish parliament began drafting new national structures and processes for a foresight-linked AG [55, 56]. Since then, Finland’s foresight capability and future-oriented policies have served as an exemplary model for other countries [57–62]. Under the umbrella of “visionary politics,” Finland utilizes various foresight programs to make policies and decisions [61, 63]. It means that Finland conducts extensive futures research through AG for the future of its state affairs. In this regard, Finland has four networked but rather independent main actors, namely, (1) administration, (2) parliament, (3) research institutions, and (4) social and academic societies. Close coordination between the Prime Minister’s Office (PMO) foresight functions the Parliamentary Committee for the Future (PCF), other research and academic institutes, and interest groups utilizes foresight results and applies them to a policy-making and relevant future-oriented programs. These actors cooperate and sometimes challenge one another over the implementing of foresight and feedback processes ultimately sustaining AG. Table 3 below summarizes the analysis of Finland’s AG under the proposed new framework.

**Table 3:** Finland’s AG level under the new framework

Criteria	Finland capability and system
Foresight system	- Periodic government foresight reports by the Prime Minister’s Office - Government Foresight Group organized by ministers [But] The government’s role concerning results of the public discussion is rather limited.
Networked system	- National Foresight Network with workshops, events, and expert discussions - Ministry- and regional-level coordination to facilitate a policy integration - Open discussion for non-commercial societies and research/academic institutions
Feedback system	- Independent actors including the government, which challenge and overwatch each other - Review by the Parliamentary Committee of the Future on a foresight report - Private-side evaluation on foresight process and policy integration
Continuity system	- Implicit consensus on foresight implementation and a future-oriented culture - Voluntary foresight through a venue for participation - Repetitive practices with various types of foresight projects - Foresight training/education from academic institutions

The foresight unit of the PMO issues the Government Report on the Future on a 4-yearly basis (each electoral period) [64]. The PMO usually defines targets and common themes and coordinates foresight activities with experts in the country’s ministries. The Prime Minister also leads and appoints a minister group, namely, the Government Foresight Group, which is responsible for leading national joint foresight activities with relevant stakeholders and making this operation visible [60]. They produce 12 sectoral future reviews to examine issues based on their ministries’ perspectives and capacities. If this review process proves the applicability and feasibility of a foresight outcome, the implementation is facilitated, and the project launched [65]. In addition, the PMO and the ministers’ group together provide spheres for futures-oriented discussions, but the role concerning the results of the discussions has been rather limited.

The PCF connects private, informal, and nongovernmental societies to take part in foresight projects and review government future reports, and functions as a place for anticipatory learning (e.g., as an education and training venue). First, the PCF creates networks for Finland’s forward-looking discussions and systemic crowd sourcing from the public to devise its own foresight projects (e.g., 2009 Government Foresight Report on Long-term Climate and Energy Policy [66], the Radical Technology Inquirer project launched by the PCF in 2012 [67]). Active futures-oriented PCF members facilitate and impact public discussions concerning various foresight themes. Second, during the foresight planning stage, the Committee reviews the first version of the reports on behalf of the entire parliament [63]. Moreover, along with reviews by both the PMO and private expert groups, the PCF evaluates again the effectiveness of the preparation processes a year after the approval of the reports [59]. Third, the PCF has been an arena where typically young or newcomers in politics have orientated their future-oriented attitudes and learned how to cooperate with other parliament members, especially future-oriented seniors. The PCF works on a consensus basis, in order to support its members to develop a common view of future possibilities.

A major strength of Finland’s AG system remains in its networking actors. In a country of around 5.5 million people, 700 members both from public and private sectors represent the country’s futures-oriented interest groups. These future-oriented, non-formal, and non-commercial societies and research institutions attempt to impact national decision-making. Sometimes, they compete with one another but ultimately discuss the better future of Finland. First, the administration along with the Finnish Innovation Fund (SITRA) operates the National Foresight Network and conducts monthly meetings and Foresight Friday gatherings [57]. It aims at “raising awareness of Finland’s new challenges and opportunities so they can be discussed, studied, and considered in decision-making” [68]. Second, various research and academic institutions such as Finland Futures Research Centre, the Academy of Finland, SITRA, Futures Studies Society of Finland, and the FinnSight network coordinate and implement various foresight projects in a joint manner [60–62]. Their repetitive foresight practices provide an opportunity to enhance the future fluency of professionals and future literacy for the public. Besides, foresight is an important side activity for many institutes, e.g., Business Finland finances innovation activities of Finnish firms.

#### AG of the UK and the Netherlands: lessons learned

Horizon scanning activities of both the UK and the Netherlands seem to have disappeared with little trace [69]. The horizon scanning programs in both countries were established as a core activity for a foresight-linked AG, but were abandoned by both governments. The academic literature regards both countries as exemplary models, but in reality, few such activities and applications to policies have been achieved since 2012. In particular, the COVID-19 pandemic has triggered much criticism toward the UK government which has long neglected warnings from horizon scanning and insisted on “conclusive evidence-based” policymaking instead [70, 71].

In the UK, the Horizon Scanning Centre was established in 2005. Despite also having founded the Foresight Programme, they both analyzed the futures domain [69]. The UK then introduced horizon scanning which can be used by all departments and agencies [72]. At the beginning, horizon scanning activities in the UK focused on a classical, wide-spectrum analysis of all economic sectors and promising technologies (Delta Scan) and then evolved to more focused approaches such as intelligent infrastructures, tackling obesities, and flood and coastal defense (Sigma Scan) [19, 69]. Unlike a foresight practice that looks at big issues 20–100 years into the future, the UK horizon scanning was made available to discrete issues among the entire spectrum of public policies 10–15 years into the future [73]. To meet the diverse demands of public policies, UK promoted cross-departmental activities while facing multi-disciplinary challenges proactively. It also emphasized the importance of fostering a future-oriented attitude and culture among government officials. In March 2014, the Horizon Scanning Programme Team was created after the Cabinet Office’s Horizon Scanning Secretariat merged with the Government Office of Science’s Horizon Scanning Centre [20].

The distinctive features of the UK’s horizon scanning were about feeding evidence-based policies into each department and its decision-making [22]. The UK horizon scanning relied on robust evidence and well-designed, conclusive studies [71]. It was inevitable that its scanning drew upon the S&T bases because the UK’s scanning function had been under the Office of S&T [72]. These scanned outcomes were used as basic information, indicators, and a scoping device for specific strategic and forward-looking functions such as scenario building, technology assessment, and strategic foresight. The outcomes also supported an appraisal of the long-term agenda residing across the government. It was for the government to test the resilience of the policy drafts of each department [22]. However, an increasing number of scientists within the current civil service system jeopardized the transformation of horizon scanning into a future-oriented process that allows “unasked questions” to be asked or the “unknown unknown” to be identified. The UK’s horizon scanning started to have a distance from systematic and imaginative analysis of trends, risks, and possibilities around the government.

The UK’s horizon scanning also maintained fairly fragmented project operations. It means that the scanning authority’s core responsibility was to coordinate the scanning activities of different departments that would otherwise implement them in a fragmented way [69]. The fragmented approach somewhat allowed for more rapid scanning and filled a specific gap in government officials’ understanding of policies. However, this fragmentation resulted in less in-depth scanning activities and the duplication of departmental efforts [72]. These rather narrow and duplicated outcomes did not provide a reasonably objective foundation for the necessary political process that leads to implementable policies. Often lengthy and poorly presented scanned output does not translate well for ministers and senior officials. Decision makers, usually enduring busy daily lives, often have difficulty in finding time to engage with the output which might not impact anything for up to 50 years [74]. In other words, it is easier to ignore.

When implementing horizon scanning activities, there was weak integration among UK departments, particularly between the decision makers and participating stakeholders [74]. The UK had an extensive network of participating actors for horizon scanning. The Horizon Scanning Centre was the networking venue for the Foresight Directorate, the Research Councils, the Government’s Chief Scientific Adviser, the Prime Minister’s Strategy Unit, the Future Security and Intelligence Outlook Network, and the Departmental Chief Scientific Advisers [72]. There were communities of interest such as the Horizon Scanning Private Sector Network, Global Futures Groups, and a network of future analysts (FAN Club) as well [19, 20]. However, they were not centrally coordinated and managed by the Horizon Scanning Centre. (This malfunction triggered the above institutional merges in 2014.) This lack of integration was due to departmental silos which make scanning unsystematic [72]. These silos also meant that including outside sources of information and expertise became difficult.

Despite many foresight activities having been done through horizon scanning nationwide over the last 20 years, UK horizon scanning is only now evaluated as having “no comprehensive understanding across government as a whole of the future risks and challenges facing the UK” ([75] , p.3). The scanning project only lasted for around 2 years, with an addition year for evaluation [69]. On paper, the UK seems to have good foresight and horizon scanning functions. However, in reality, the administration does not value sufficiently preparing for the future while lacking in coherence in planning which ultimately risks misinterpretation for future policymaking [76]. The analysis of the UK’s AG based on the newly proposed framework is listed in Table 4 below.

**Table 4:** The UK’s AG level under the new framework

Criteria	UK capability and system
Foresight system	- Foresight projects by the government’s Foresight Programme - Horizon Scanning Programme Team to feed the evidence-based policies [But] The heavy reliance on robust evidence and well-designed jeopardizes transformation of horizon scanning to a future-oriented process that allows to identify “unknown unknown”.
Networked system	- Horizon Scanning Programme Team as a central coordinating body [But] The UK horizon scanning is fairly fragmented, and strong department silos have a scanning not systemic and make difficult departments to integrate. - Policy debate, roundtable discussion, workshop with different stakeholders - Many communities of interest including the Horizon Scanning Private Sector Network [But] The above silos make difficult to include outside information and external expertise.
Feedback system	- Evaluation of the reports and policies through extranet, peer review, discussion groups - Workshops devoted to test departments’ strategies on resilience with the scanned output [But] The projects only lasted for around 2 years with further 1-year follow-up despite it was four or five to be underway.
Continuity system	- Many repetitive foresight and horizon scanning projects since the establishment of UK Foresight Programme in 1994 [But] It disappeared with little trace since 2012. - Foresight academic institutions: Manchester Institute of Innovation Research, the University of Sheffield’s Regional Technology Foresight, Oxford Martin School

The Netherlands established the Commission for Consultation of Sector Councils (COS) in the 1980s. The COS was created to connect the government, researchers, and societies while identifying and formulating strategic knowledge relevant to Dutch policy domains and their departmental policies [19]. In 2007, it disappeared and changed to the “knowledge chambers,” whose horizon scanning function was allocated to the newly formed Knowledge Directorate of the country’s Ministry of Education, Culture, and Science [19]. Recently, horizon scanning in the Netherlands is largely implemented as a part of the European Union’s horizon scanning activities.

In 2004, the COS initiated national horizon scanning activities which seek to identify future problems, threats, and opportunities in all policy domains [19]. Similar to the UK, the role of the horizon scanning body in the Netherlands was to facilitate cross-departmental scanning activities and overarching studies that include as many societal domains as possible [19, 20]. The main objective of the scanning was to make the Netherlands more sustainable and its policymaking more resilient. Its horizon scanning supported the framing of policies in a more future-oriented manner by scanning over the wider societal dimensions (environment, energy, health, longevity, welfare, human rights, integration of society, law-based democratic society, foreign affairs, etc.) [19]. In addition, scanned outputs were utilized as the foundation of the strategic planning and prioritizing of research by the National Funding Agency [19].

Based on the COS’s focus on participative foresight, a wide range of participation was encouraged in Dutch horizon scanning activities such as the Horizon Scan Report 2007 and Horizon Scan 2050. Various networking bodies including an advisory group composed of government officials from ministries and internal workshop with employees shaped the scanning processes [20]. All stakeholders including the public had an opportunity to suggest new issues and comment on government-suggested concerns through an open internet site [19]. For example, the government uploaded the scanned output at an early stage of the project and then received the stakeholders’ opinions regarding the plausibility and impacts of the policies. Above all, its unique issue-based clustering approach for assembling potentially impact-rich interactions of policies was supported by creative group-thinking exercises.

The Netherlands’s horizon scanning in 2007, namely, the Horizon Scan Report 2007, revealed various issues. While its time horizon was very long term, its policy focus was to identify important shorter-term issues that needed to be incorporated in the current policy agenda for a period from 2 to 3 years [77]. It embedded the danger of focusing too much on near-term issues while neglecting to explore novel and unexpected ones. Moreover, similar to the UK, the decision makers downgraded the importance of scanned outputs because of their lack of depth which was caused by insufficient resources to deepen the most alarming issues [77]. Present-day policymaking in the Netherlands tends to rely more on model-based forecasting, and not on horizon scanning data.

In 2012, the Netherlands Study Centre for Technology Trends (STT) implemented a foresight project called Horizon Scan 2050 [78]. Unlike in 2007, the STT chose a broad perspective and a specified long perspective up till the year 2050. It also applied a clustering approach, namely, those of a thematic nature such as gaming, information technologies, and labor [78]. The Horizon Scan 2050 used all levels of formal and explicit techniques including workshops, expert views, and desk research. It attempted to achieve a creative process of collective sense-making through gathering and synthesizing observations [21]. However, the Netherlands’s own national horizon scanning no longer exists alone and belongs to the European Union’s horizon scanning activities. Analysis of the level of the Netherlands’s AG based on the proposed new framework is listed in Table 5.

**Table 5:** The Netherlands’s AG level under the new framework

Criteria	The Netherlands capability and system
Foresight system	- Two planning bureaus including the Central Planning Bureau in charge of horizon scanning [But] The present-day policy-making tends to rely more on evidence-based forecasting for each issue cluster, not the horizon data. It means scanned outputs tend to be short-sighted while neglecting to explore novel and unexpected issues.
Networked system	- Internal workshops with experts and advisory group composed of the government officials from ministries - Citizen and stakeholder’s participation on project through workshops and open internet site - Issue clusters facilitated by creative group thinking exercises
Feedback system	- Networked bodies as an evaluator and reviewer during the project implementation [But] It is hard to find feedback system after a project is finished and the final is released.
Continuity system	- Two published major reports of horizon scanning. - [But] Since 2012, no scanning report has been published by the Dutch government alone. - Now, most of horizon scanning functions of the Netherlands are as a part of the European Union’s horizon scanning activities.

#### Korea’s AG: re-starting after a 40-year-long deadlock

The unique feature of Korea’s AG is that its foresight and AG underwent a period of deadlock for 40 years. Foresight was first introduced in 1968 by the Year 2000 Committee, while foresight outcomes were used as a theoretical basis to drive economic and industrial development policies [79]. After the Korean War, the Korean political and economic situation assumed Western-styled modernization as the desired “one future” [79–81]. Building national competitiveness including wealth and strong military power was set as the nation’s utmost vision and top policy priority. All economic and political policies were designed to overcome postwar poverty and the political and ideological confrontation between North and South Korea. Therefore, it was inevitable that a foresight-linked AG would go through an assimilation process with a “development agenda”. It is understood as national short-term planning in order to implement an “economic growth first” agenda.

Despite all the above, Korea intermittently implemented foresight projects. As a form of long-term planning and strategy, research institutes under the strong leadership of each regime prepared and published foresight reports. For example, after the Asian Financial Crisis in 1997, foresight policy integration started to gain traction in government affairs and various agendas [79, 82]. The milestone foresight report written by Alvin Toffler in 2001 was prepared and implemented inter-ministerially and region wide under strong leadership and an orientation to action the presidential office [83]. Toffler’s foresight known as “knowledge-based society” was fully integrated into the national vision and policies. Having this initiation, various foresight-linked AG institutions were established [79]. In the administration, the Long-Term Strategy Bureau in the Ministry of Strategy and Finance in 2012, the Ministry of Information and Communication’s Future Strategy Office in 2005, and the Secretary for the Future in the President Senior Secretary for the National Planning Office in 2008 all conducted foresight activities. Committees including the Presidential Council for Future and Vision in 2008, the Research Committee of Future & Fusion in the PMO in 2014, and the Future Preparation Committee in the Ministry of Science and Technology (S&T) in 2013 were also assembled for greater policy integration. However, it was mostly implemented in the middle of each administration’s office period and on needs basis of the government [84]. In short, the feature of foresight policy integration in Korea could be described as government-led, unilateral national planning and policymaking. Analysis of the level of Korea’s AG based on the proposed framework is listed in Table 6.

**Table 6:** Korea’s AG level under the new framework

Criteria	Korean capability and system
Foresight system	- Foresight reports produced from research institutions [But] They were intermittently published on a needs basis of the government and tainted as a decoration to political agenda and economic plans. - A recently established National Assembly Futures Institute (NAFI) in 2013 [But] It is still in infancy so many limitations still exist.
Networked system	- Strong commitment of leadership and highly centralized structure [But] Instead of being networked, Korea rather has a government-led unilateral foresight. - Various high-level foresight entities in various ministries and bureaus [But] They are fragmented and quickly dis-established by political needs and changes.
Feedback system	- Expert review and committee’s formal evaluation process on foresight report [But] It is also standard process for all government reports, not just for foresight tasks. - Regular parliamentary inspection [But] There is no parliamentary review based on a future-oriented attitude*.
Continuity system	- Its early initiation of the AG in 1968 [But] Korea experiences the 40-year-long deadlock of foresight and the AG until 2013 - Government-approved education institutes established in 2012 - Mandatory foresight training for all government officials in 2020 [But] It is still in infancy so many limitations still exist.

*In 2001, the Special Committee for Future Strategy and S&T was established to support evaluations of ministries’ long-term planning and strategy. However, due to its temporal standing position, the committee did not fully exercise its power on providing feedback and building routine AG process and quickly disestablished

Amid this lack of vividness in terms of AG, led by a few futures researchers and futures-oriented key figures, a significant leap-forward has been identified recently. In 2013, as the first government-approved foresight and futures studies institution based on S&T, Korea Advanced Institute of Science and Technology (KAIST) opened the Graduate School of Future Strategy in Korea’s administrative capital Sejong to educate and train professionals in foresight with advanced academic degrees [79]. Since 2013, foresight has started to gain in social, economic, and political importance within government affairs especially policymaking. In 2018, as the most comprehensive foresight-linked AG coordinating body, the National Assembly Futures Institute (NAFI) was finally established in the Korean National Assembly [79]. Taking the SITRA and the PCF of Finland as a role model, in 2018, NAFI was established to be an exclusive, competitive, permanent, overarching, neutral, and participatory foresight research institute of AG [85, 86]. NAFI is to implement national foresight on a regular basis and then manage and control research outputs for the whole country under the AG scheme.

### Summary of the comparison

Based on the above comparative analysis, this article identifies that the consequences of a foresight-linked AG are different in each country. Finland has continuously implemented foresight in government affairs. Unlike Finland, however, the UK and the Netherlands have not actively utilized their national horizon scanning activities since 2012. Indeed, Korea is a unique case in this regard. Despite its early start in foresight in the 1960s, Korea did not properly implement a foresight-linked AG. However, recently, Korea has re-started its national foresight to achieve AG through establishing a foresight-dedicated research institute in 2018, the NAFI.

The AG level of each country reveals distinctive implications. First, Finland values its public’s opinion, and their participatory input can overwhelm the government’s influence. For example, the PMO cannot influence the results of a public discussion. Moreover, the PCF works as an education and training venue for young politicians where they learn the values of a foresight-linked AG and participate in a forward-looking manner in their early careers as civil servants. Second, UK did not internalize a forward-looking attitude within the administration where it is rather fragmented while hasty implementation of horizon scanning inevitably lowered the quality of scanned outcomes, resulting in a less in-depth practice. Due to this, these outcomes did not properly translate into the “language” of the government’s decision makers. No matter how the public and various stakeholders insist on scanning outcomes, the government officials could not place any high value on the importance of the horizon scanning activities. Therefore, the UK government could not properly apply foresight outcomes in the present policy agenda and governmental decision-making processes. Third, for the Netherlands, despite outstanding horizon scanning projects, it is hard to find that the scanned outcomes actually influenced decision makers. The scanned outputs were short-sighted and neglected to explore novel and unexpected issues. Fourth, the history of Korea explains that a foresight-linked AG was treated within the context of a set political agenda with short-term administrative plans and governmental strategies for development. Moreover, the public and scholars from other traditional administrative and academic domains were not ready to accept the values of forward-looking activities and future-oriented minds.

## Discussion

### *Future receptivity*, a forward-looking capacity of “human or people”

The framework-based analysis identifies a common denominator on how to achieve a successful AG and to prevent AG from obsolescent and misinterpretation. It is critical to determine success or failing factors of AG in governmental affairs. The results in a sense explain that all countries appear to meet the basic criteria to some degree. For example, they have (or had) a foresight system including projects and organizations, a system for communication and participation, a feedback system for evaluating and reviewing foresight outcomes, and a certain degree of an education and training system. However, this comparative analysis identifies a very delicate difference, *future receptivity*: a forward-looking capacity of ‘human or people. It may be subtle, but important to countries seeking to start a foresight-linked AG.

For the UK, the Netherlands, and Korea, systems and structures came first before receptivity, a “human or people” capacity to accept and understand the values of foresight. Due to the lack of forward-looking training or education in future-oriented attitudes, decision makers in the government still seek “hard evidence”. Consensus from the public and insights from experts such as scholars and researchers are hardly acceptable. Their lack in *future proficiency* makes them reluctant to engage in anticipatory learning which then leads to short-sightedness. In addition, the public do not have sufficient knowledge about AG. Their low *future literacy* renders them weak when trying to impose their opinions on the policy agenda. It was hard for the public who do not know about the meaning of foresight, to argue with decision makers and reiterate images and view of their “desired future” to policymaking.

### Implications for newcomers

Each country has a different cultural context, social systems, and political as well as organizational structures. It means that an application of a specific institution or certain system such as a foresight-linked AG which is successfully implemented in one country to other countries is relatively hard. Therefore, for newcomers, a simple but comprehensive and fundamental theme should be suggested in order to achieve AG within an existing system and structure. Taking into account *future receptivity* mentioned above, two general proposals to initiate and continue a foresight-linked AG are suggested to newcomers, specifically how to build *future receptivity* for long-term thinking and how to get foresight-linked AG out of the short-term interest sphere.

Newcomers should first set up a mandatory public participation process (or venue of networking) while granting the public power to review and change foresight outcomes. The needs-based and public-driven policymaking grants a government legitimacy to implement foresight outcomes [87, 88]. Governing bodies should include the public at the initial foresight production stage such as implementing a mandatory survey of the public for a country’s long-term plans, development strategies, and even policy-related reports. To facilitate this inclusion and to make this participation easy, it is necessary to establish a nationwide foresight network for the public. This network can build an “anticipatory” culture within societies similar to that of Finland. Increased societal demands for foresight can then push government officials to think openly and liberally, consider alternative futures and strive for a creative vision. It means a country’s decision makers including government officials cannot free themselves from the public influence. Public participation in foresight processes allows policy makers and government officials to understand demands as well as future needs of the public. Strong public influence provides continued feedback, and public participation can be a safeguard against an administration’s unilateral decisions in long-term planning. In short, it plays a check-and-balance role [89, 90]. In addition, a participatory network can strengthen the public ownership of foresight outputs which demands foresight implementation and pushes the application of AG into the sphere of government affairs. Therefore, public participation is at the core of foresight processes and supports sustainable operations of AG.

Both government officials (including those at the senior level) and the public need to understand the values of AG and processes of foresight through education and training. For professionals including government officials, its receptivity is known as *future proficiency,* a higher level of *future receptivity*. Thinking about future generations and governing a country to them is the responsibility of government actors. It is because the domains of futures are similar to “public goods,” which are not automatically provided by individual actors in society. It means that as an initiator of a foresight-linked AG and a facilitator for the public, government officials need to fully understand the meaning of foresight. Taking into account the lessons from Finland, repetitive field practices can foster *future proficiency* for the professionals. Field practices of foresight and AG, including government-funded projects, can integrate participants including decision makers, scholars, and government officials and ultimately increase the practical applicability of foresight to policymaking. Continued practices shape a foresight-friendly environment within a government and its administration and can become a strong foothold for policy implementation processes. Moreover, the inter- or cross-disciplinary nature of foresight practices can be a great venue for interdisciplinary research that embraces various academic domains under the value of futures while building a coordinating and learning network. Foresight-linked AG practices and projects can become a catalyst to internalizing a foresight capability for government officials and to distribute knowledge of foresight to other academic domains. This is especially so by including all level of stakeholders including private experts and scholars; they can have an opportunity to practice and apply foresight into real-world cases.

Based on the aforementioned public participation, trained and educated government officials need to include the public in all future-related practices, projects, decision-making, and policymaking. It is almost impossible that the public as a whole is being educated and trained by a certain program or a system directly. It is more about exposure and experience, namely *future literacy*. As mentioned above, through various participatory processes such as public hearings, citizen panels, survey, and participatory foresight methods (e.g., future cafés, futures workshops), professionals can increase the level of foresight exposure to the public so they can expand their knowledge horizon. The public can be empowered with “anticipatory” and forwarding-looking capacities [29, 32, 89]. If *future literacy* is well nurtured, a knowledgeable public can demand from the government long-term policy planning rather than the risk-averse tendency.

Education and training of both government officials and the public needs to be done together. It is because the public is the final national policy consumer. Public participation in foresight-linked AG processes allows policymakers and government officials to understand the demands of the public. It means both parties should communicate effectively on the foresight agenda and build a consensus in a future-oriented manner. This forward-looking and participatory process can lead foresight-linked AG to produce a distinctive, creative, and qualified long-term outcome [91, 92].

## Conclusion

The newly proposed framework is applied as a diagnostic tool for evaluating the AG levels of Finland, the UK, the Netherlands, and Korea. This framework shows where and how certain approaches of a country may need improvement for a successful and continued foresight-linked AG. Based on the above comparative case analysis, each analyzed country shows its own modality and consequences of AG while these countries also share several similarities in implementing foresight and AG. However, their different levels of *future receptivity* within the public and government officials determine a continuity of foresight and the sustainable operation of AG. The individual capacity to understand and accept the value of foresight needs to be considered greater in the development of a foresight-linked AG. Therefore, it is important for newcomers to foster *future receptivity*. First, a government should educate and train the public and government officials to promote future literacy and future proficiency. Meanwhile, a government should provide an effective process of public participation while granting them power to influence their diverse future images on a foresight outcome.

Unlike Fuerth and Farber, this article focuses on a permanent and long-term approach—emphasis is on the “human or people” ranging from the public to the highest national decision makers. It is because futures studies is the academic backbone of foresight and AG, which values various futures within both society and people and encompasses changing variables [1, 12, 14, 17, 93]. It means the methods, processes, and approaches of a foresight-linked AG should be open and flexible for long-term continuity. AG cannot be achieved with any temporary system or organizational modification. A continued driving force including human capability and public participation enables foresight to be initiated and AG to be maintained in a sustainable way. A future-oriented attitude, thinking, and knowledge should be ready before making any adjustment or systemic upgrade in a government system.

These days, countries including the UAE, India, Thailand, Russia, and South Africa have attempted to introduce AG into their government affairs [1–5]. If these efforts are based on a relative minimum adjustment of the decision-making structure and small changes in the governance system, these newcomers’ aspirations to AG cannot be achieved or sustained. Including Korea, these newcomers are now being pushed to play a role as a creative leader by international societies. Future uncertainties and rapid societal changes require them to amend their stances to be more forward-looking and future-oriented. They now face emerging needs of their own future model. Promoting *future receptivity* will be a good starting point for them to build their “desired” future.

## Data Availability

Not applicable

## Abbreviations

AG: Anticipatory governance
COS: Commission for Consultation of Sector Councils in the Netherlands
NAFI: National Assembly Futures Institute in Korea
KAIST: Korea Institute of Public Administration and Korea Advanced Institute of Science and Technology
PMO: Prime Minister’s Office
PCF: Parliamentary Committee for the Future in Finland
SITRA: Finnish Innovation Fund
STT: Study Centre for Technology Trends in the Netherlands
S&T: Science and Technology
UAE: United Arab Emirates
UK: United Kingdom
US: United States

## References

[1] Fuerth LS, Faber EM (2012). Anticipatory governance practical upgrades: equipping the executive branch to cope with increasing speed and complexity of major challenges.

[2] Future foresight: shaping the future. (2017) United Arab Emirates Ministry of Cabinet Affairs & the Future. https://www.mocaf.gov.ae/en/area-of-focus/future-foresight. Accessed Dec 24, 2018

[3] Karuri-Sebina G, Rosenzweig L (2012). A case study on localising foresight in South Africa: using foresight in the context of local government participatory planning. Foresight.

[4] Calof J, Smith Jack E (2012). Foresight impacts from around the world: a special issue. Foresight.

[5] Dreyer I, Stang G (2013). Foresight in governments–practices and trends around the world.

[6] Kim Y, Lim S, Park J, Yoon G, Tak H, Jung S, Ji G (2019). Anticipatory governance and government innovation.

[7] Foresight manual - empowered futures for the 2030 Agenda (2018). UNDP Global Centre for Public Service Excellence, Singapore

[8] Office for Government Policy Coordination (2019). Result Report of Participating 38th Session of the Network of Senior Officials from the Centres of Government.

[9] Fuerth LS (2009). Foresight and anticipatory governance. Foresight.

[10] Boston J (2016) Anticipatory governance: how well is New Zealand safeguarding the future? Policy Q 12(3). 10.26686/pq.v12i3.4614

[11] Calof J, Smith JE (2010). Critical success factors for government-led foresight. Sci Public Policy.

[12] Dator J (2019). Alternative futures at the Manoa School.

[13] Inayatullah S (2002). Reductionism or layered complexity? The futures of futures studies. Futures.

[14] Marien M (2002). Futures studies in the 21st century: a reality-based view. Futures.

[15] Coates JF (1985). Foresight in federal government policy making. Futur Res Q.

[16] Martin BR (1995). Foresight in science and technology. Tech Anal Strat Manag.

[17] Slaughter RA (1996). The knowledge base of futures studies as an evolving process. Futures.

[18] Schultz WL (2006). The cultural contradictions of managing change: using horizon scanning in an evidence-based policy context. Foresight.

[19] van Rij V (2010). Joint horizon scanning: identifying common strategic choices and questions for knowledge. Sci Public Policy.

[20] Cuhls KE (2020) Horizon scanning in foresight – why horizon scanning is only a part of the game. Futur Foresight Sci 2(1). 10.1002/ffo2.23

[21] Könnölä T, Salo A, Cagnin C, Carabias V, Vilkkumaa E (2012). Facing the future: scanning, synthesizing and sense-making in horizon scanning. Sci Public Policy.

[22] van Rij V, in’t Veld RJ (2010). Horizon scanning: monitoring plausible and desirable futures. Knowledge democracy: consequences for science, politics, and media.

[23] Popper R (2008). How are foresight methods selected?. Foresight.

[24] Könnölä T, Scapolo F, Desruelle P, Mu R (2011). Foresight tackling societal challenges: impacts and implications on policy-making. Futures.

[25] Foresight - the manual (2014) UNDP Global Centre for Public Service Excellence, Singapore

[26] Bevir M (2012). Governance: a very short introduction.

[27] Kooiman J (1999). Social-political governance. Public Manag.

[28] Rhodes RAW (1996). The new governance: governing without government. Pol Stud.

[29] Poli R (2017). Introduction to anticipation studies.

[30] De Jouvenel B (2017). The art of conjecture.

[31] Fuerth LS (2013). Anticipatory governance: winning the future. Futurist.

[32] Guston DH (2014). Understanding ‘anticipatory governance’. Soc Stud Sci.

[33] Osborne D (1993). Reinventing government. Pub Prod Manag Rev.

[34] Quay R (2010). Anticipatory governance. J Am Plan Assoc.

[35] Misra DC, Hariharan R, Khaneja M (2003). E-knowledge management framework for government organizations. Inf Syst Manag.

[36] Bertot JC, Estevez E, Janowski T (2016). Digital public service innovation: framework proposal. Paper presented at the 9th International Conference on Theory and Practice of Electronic Governance, Montevideo, Mar 1-3, 2016.

[37] Farooque M (2011). Dhaka megacity: from reactive government to anticipatory governance. Paper presented at the BAPA-BEN urbanization conference, Dhaka, Jan 8, 2011.

[38] DeLeo RA (2017). Anticipatory policymaking in global venues: policy change, adaptation, and the UNFCCC. Futures.

[39] Bächler G, Austin A, Fischer M, Ropers N (2004). Conflict transformation through state reform. Transforming ethnopolitical conflict: the Berghof handbook.

[40] Lundy Dobbert M (2000). Anticipatory anthropology and world peace: a view from 2050. Futures.

[41] Hakken D (2000). Resocialing work? Anticipatory anthropology of the labor process. Futures.

[42] Barben D, Fisher E, Selin C, Guston DH (2008). Anticipatory governance of nanotechnology: foresight, engagement, and integration. The handbook of science and technology studies.

[43] Davies SR, Selin C (2012). Energy futures: five dilemmas of the practice of anticipatory governance. Environ Commun.

[44] Bezold C (2006) Anticipatory democracy revisited. Democracy Futures:38–51

[45] Feltmate BW (1993). Barriers to achieving sustainable development in North America: historical naivety, media limitations and non-anticipatory governance.

[46] Boston J, Wanna J, Lipski V, Pritchard J (2014). Future-proofing the state: managing risks, responding to crises and building resilience.

[47] Kümpers S, Mur I, Maarse H, Van Raak A (2005). A comparative study of dementia care in England and the Netherlands using neo-institutionalist perspectives. Qual Health Res.

[48] Scharpf FW (2000). Institutions in comparative policy research. Comp Pol Stud.

[49] Yin RK (2017). Case study research and applications: design and methods.

[50] Herriott RE, Firestone WA (1983). Multisite qualitative policy research: optimizing description and generalizability. Educ Res.

[51] Ragin CC (2014). The comparative method: moving beyond qualitative and quantitative strategies. 1st with a new introduction edn.

[52] Ragin CC, Amoroso LM (2011). Constructing social research: the unity and diversity of method.

[53] Chi KS (2008). Four strategies to transform state governance. Organizational Transformation Series.

[54] Ramos JM (2014). Anticipatory governance: traditions and trajectories for strategic design. J Futur Stud.

[55] European Environmental Agency (2011). Finland Country Case Study. Annex 2, vol EEA Technical Report.

[56] Organization for Economic Co-operation and Development (2005). Territorial review of Finland.

[57] Kaivo-oja J, Marttinen J, Varelius J (2002). Basic conceptions and visions of the regional foresight system in Finland. Foresight.

[58] Kaivo-oja J, Marttinen J (2008). Foresight systems and core activities at national and regional levels in Finland 1990–2008.

[59] Heinonen S, Lauttamäki V (2012). Backcasting scenarios for Finland 2050 of low emissions. Foresight.

[60] Ahlqvist T (2015). Foresight activities in Finland: actors, relations, and impacts on policy making. Paper presented at the 6th International Conference and Workshop, Tokyo, March 2-4, 2015.

[61] Nováky E, Monda E (2015). Futures studies in Finland. Soc Econ.

[62] Tapio P, Heinonen S (2018). Focused futures from Finland. World Futur Rev.

[63] Tiihonen P, Zsolnai L, Boda Z, Fekete L (2009). The right of future generations. Ethical Prospects. Economy, society, and environment.

[64] Finland Prime Minister’s Office (2018). Government report on the future.

[65] Finland Prime Minister's Office (2018). The ministries’ joint foresight activities.

[66] Finland Prime Minister’s Office (2009). Government foresight report on long-term climate and energy policy: towards a low-carbon Finland. Government foresight report, vol 30.

[67] Vasamo A-L (2015). The Radical Technology Inquirer (RTI) tool for technology anticipation and evaluation: introduction and quality criteria analysis. Eur J Futur Res.

[68] Paukkunen S (2017). Foresight and futures work in the Finnish Government. Paper presented at the Meeting on foresight Activity between Finland and Korea, Seoul, April 27, 2018.

[69] Miles I, Saritas O (2012). The depth of the horizon: searching, scanning and widening horizons. Foresight.

[70] Majeed A, Seo Y, Heo K, Lee D (2020). Can the UK emulate the South Korean approach to covid-19?. BMJ.

[71] Rose D, Burgman M, Sutherland W (2020). The civil service doesn’t just need more scientists – it needs a decision-making revolution. LSE Impact of Social Sciences, vol 2020.

[72] Schmidt John M (2015). Policy, planning, intelligence and foresight in government organizations. Foresight.

[73] Glasspool M (2011). The UK foresight programme. Paper presented at the NISTEP Conference, Tokyo, March 8-9, 2011.

[74] Day J (2013). Review of cross-government horizon scanning. 2010 to 2015.

[75] Leadership for the long term: Whitehall’s capacity to address future challenges (2015). The Stationery Office Limited, Longdon

[76] Dixon J (220) The endemic failure of long-term policymaking. Covid-19 is exposing holes in the government’s ability to plan for the future. Prospect, London

[77] Amanatidou E, Butter M, Carabias V, Konnola T, Leis M, Saritas O, Schaper-Rinkel P, Van Rij V (2012). On concepts and methods in horizon scanning: lessons from initiating policy dialogues on emerging issues. Sci Public Policy.

[78] Van der Duin P, Marchau V, Van der Goes L, Scheerder J, Hoogerwerf R, de Wilde S (2016). Challenging the future. Implications of the Horizon Scan 2050 for the Dutch Top-Industry Innovation Policy. Athens J Technol Eng.

[79] Heo K, Seo Y (2019). National foresight in Korea: history of futures studies and foresight in Korea. World Futur Rev.

[80] Scitovsky T (1985). Economic development in Taiwan and South Korea: 1965-81. Food Res Inst Stud.

[81] Kuznets PW (1988). An East Asian model of economic development: Japan, Taiwan, and South Korea. Econ Dev Cult Chang.

[82] Seo Y, Park S, Lee H (2010). Government-led futures research and future guideline of Alternative National Futures Institute. Basic Research Report, vol 21.

[83] Toffler A (2001). Overcome crisis: vision of the 21st century Korea. KISDI publication’s policy research, vol 01(08).

[84] Lee K (2015). A comparative study of national future strategy formulation in Korea and Finland.

[85] Kim ST (2013). Trend of Overseas Futures Studies Organization and establishment of National Future Strategy Center (trans: Center BDSR).

[86] Jeon S, Kim S, Kim C, Park JM, Yoon JS, Lee K, Park C, Part MH (2015). Overseas case study.

[87] Saritas O, Pace LA, Stalpers SI, Borch K, Dingli S, Jørgensen MS (2013). Stakeholder participation and dialogue in foresight. Participation and interaction in foresight.

[88] Currie-Alder B (2003). Why participation?: enhancing our understanding of participatory approaches to natural resource management; a living document for the MINGA Program Initiative (trans: Initiative MP). Living document for the MINGA Program Initiative.

[89] Tapio P (1996). From technocracy to participation?: positivist, realist and pragmatist paradigms applied to traffic and environmental policy futures research in Finland. Futures.

[90] Tonn BE (2003). The future of futures decision making. Futures.

[91] Jungk R (1973). Three modes of futures thinking. Paper presented at the Hawaii 2000: Continuing Experiment in Anticipatory Democracy, Honolulu, Jan 1, 1973.

[92] Inayatullah S (1990). Deconstructing and reconstructing the future: predictive, cultural and critical epistemologies. Futures.

[93] Dator J (2011). Stories of futures study: Korea, me and futures study.

